# Vascular smooth muscle TRPC3 channels facilitate the inverse hemodynamic response during status epilepticus

**DOI:** 10.1038/s41598-020-57733-0

**Published:** 2020-01-21

**Authors:** Michael A. Cozart, Kevin D. Phelan, Hong Wu, Shengyu Mu, Lutz Birnbaumer, Nancy J. Rusch, Fang Zheng

**Affiliations:** 1 Department of Pharmacology and Toxicology, Little Rock Arkansas, United States of America; 2 Department of Neurobiology and Developmental Sciences, University of Arkansas for Medical Sciences, Little Rock Arkansas, United States of America; 3 Neurobiology Laboratory, National Institute of Environmental Sciences, Research Triangle Park, North Carolina, United States of America; 4 Institute of Biomedical Research (BIOMED), School of Medical Sciences, Catholic University of Argentina, Buenos Aires, Argentina

**Keywords:** Ion channels in the nervous system, Neuro-vascular interactions, Epilepsy

## Abstract

Human status epilepticus (SE) is associated with a pathological reduction in cerebral blood flow termed the inverse hemodynamic response (IHR). Canonical transient receptor potential 3 (TRPC3) channels are integral to the propagation of seizures in SE, and vascular smooth muscle cell (VSMC) TRPC3 channels participate in vasoconstriction. Therefore, we hypothesize that cerebrovascular TRPC3 channels may contribute to seizure-induced IHR. To examine this possibility, we developed a smooth muscle-specific TRPC3 knockout (TRPC3smcKO) mouse. To quantify changes in neurovascular coupling, we combined laser speckle contrast imaging with simultaneous electroencephalogram recordings. Control mice exhibited multiple IHRs, and a limited increase in cerebral blood flow during SE with a high degree of moment-to-moment variability in which blood flow was not correlated with neuronal activity. In contrast, TRPC3smcKO mice showed a greater increase in blood flow that was less variable and was positively correlated with neuronal activity. Genetic ablation of smooth muscle TRPC3 channels shortened the duration of SE by eliminating a secondary phase of intense seizures, which was evident in littermate controls. Our results are consistent with the idea that TRPC3 channels expressed by cerebral VSMCs contribute to the IHR during SE, which is a critical factor in the progression of SE.

## Introduction

*Status epilepticus* (SE) is a life-threatening condition characterized by continuous or rapidly repeating seizures^1^. Attempts to understand the pathogenesis and progression of SE have historically focused on neuronal dysfunction, particularly hyperexcitation, rather than on a broader perspective that allows for the culpability of both neuronal and vascular abnormalities as contributors to SE. Blood flow is tightly coupled to the metabolic demands of local neuronal activity in a process termed neurovascular coupling, which is facilitated by highly complex interactions between neurons, glia, and vascular cells^2–5^. Brain homeostasis and proper neuronal function fully rely on intact neurovascular coupling. Accepting this tenet, it is not surprising that disruption of neurovascular coupling is increasingly recognized as a shared feature of many neurological disorders, including epileptic seizures^2,3,6,7^, and cerebrovascular dysfunction may be a key feature of SE.

Acute insults to the brain are widely reported to result in spreading depolarization^6,8–10^, or waves of neuronal depolarization emanating from the locus of the trauma and proceeding outwardly through healthy tissue. Such spreading depolarizations are observed clinically in traumatic brain injury^8,9^, stroke^9^, brain hemorrhage^6,9,10^, and epileptic seizure^6,9,10^. Spreading depolarizations have been shown to induce a transient hyperperfusion of blood flow, which is often followed by one or more periods of hypoperfusion^6,8–10^. This hypoperfusion, known as the inverse hemodynamic response (IHR), is thought to be mediated by vasoconstriction of small intracerebral arteries and arterioles^9,10^. Understandably, during the highly elevated neuronal activity of SE^11^, such periods of dysregulation favor hypoxia and hypoglycemia^6^, and by this mechanism, may contribute to the duration and severity of SE.

The canonical transient receptor potential 3 (TRPC3) channel has been increasingly implicated in SE^12–14^, and we recently reported that TRPC3 channels enhance seizure intensity^15^. TRPC3 channels are non-selective cation channels known primarily for their importance in regulating intracellular calcium. It has been known for some time that TRPC3 channels expressed by vascular smooth muscle cells (VSMCs) promote vasoconstriction via an inward cation current that further depolarizes and constricts VSMCs^16–18^. Whereas our studies implicate TRPC3 channel activation as a contributor to SE severity, other laboratories have reported that signaling pathways including G-protein coupled receptors and receptor tyrosine kinases can activate TRPC3 channels to mediate vasoconstriction^16–18^. Collectively, these findings raise the possibility that TRPC3 channels may contribute to the IHR, i.e. the pathological hypoperfusion of the brain, during SE.

In order to explore the role of TRPC3 channels in seizure-induced IHR and the progression of SE, we developed an animal model utilizing a conditional knockout of the TRPC3 channel specific to smooth muscle cells (TRPC3smcKO)^19,20^. The deletion of TRPC3 channels in this model results in a reduction of seizure-induced IHR and early termination of pilocarpine-induced SE in mice, revealing a critical contribution of TRPC3 channels and neurovascular coupling to the pathophysiology of SE.

## Results

**Figure 1:**
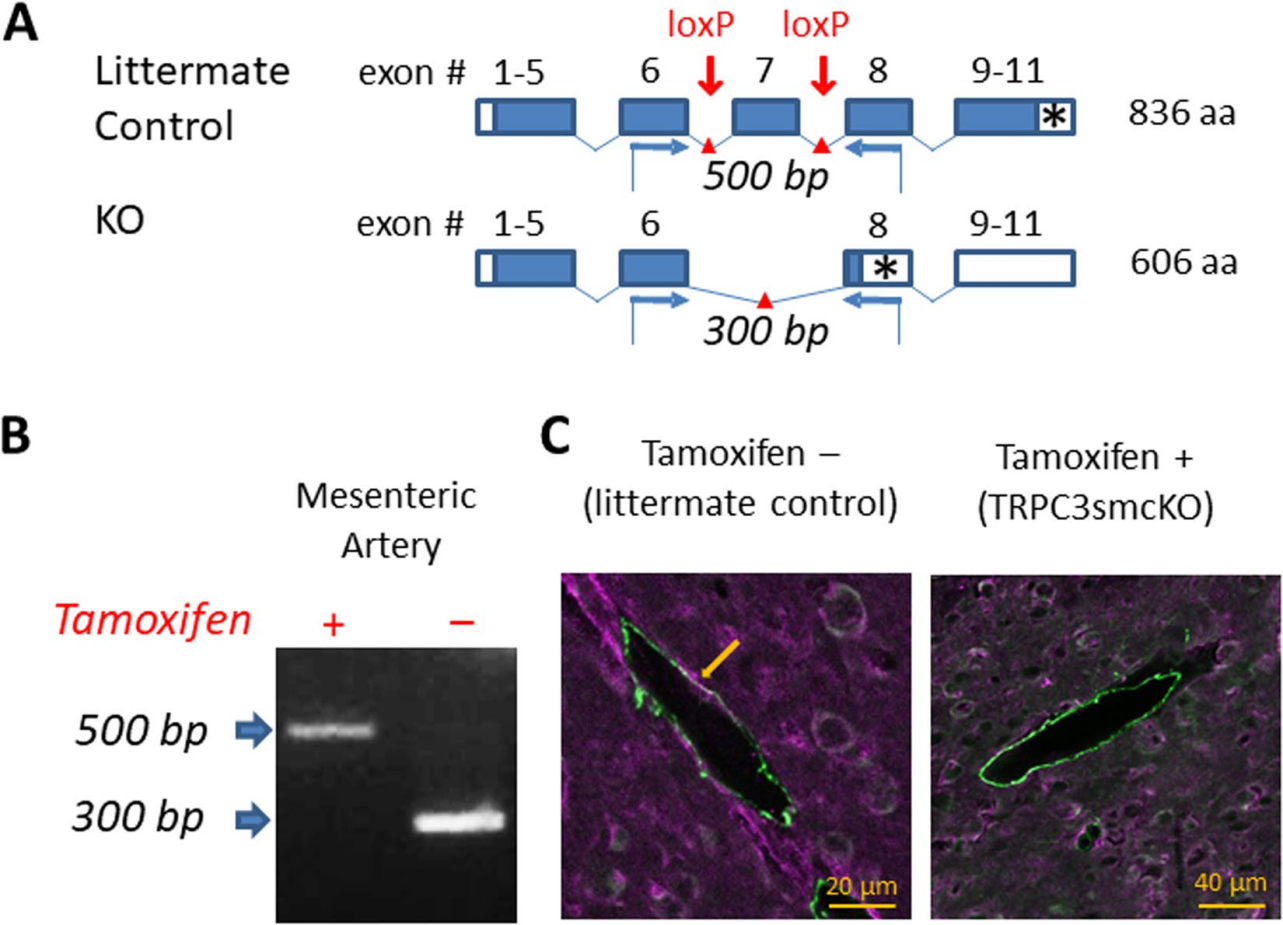
Generation of the smooth muscle-specific knock out mouse model. (**A**) Schematic illustrating strategy for TRPC3 gene deletion. Open boxes are untranslated exon sequence. Closed boxes are translated open reading frame. Asterisks denote stop codons. Red arrows mark the location of loxP sites targeting the pore-forming region (exon 7) for cleavage by Cre recombinase. Blue arrows denote forward and reverse primers for RT-PCR analysis. (**B**) RT-PCR analysis of mRNA from endothelium-denuded mouse mesenteric arteries from tamoxifen-treated (TRPC3smcKO) or vehicle-treated (littermate control) mice. 500 bp floxed amplicon of exon 7 denotes an intact exon 7 while 300 bp band indicates the cleavage fragment of exon 7 excision. No cleavage fragments were found in any tissue of untreated mice whereas only the 300 bp cleavage fragment was detected in mesenteric artery of tamoxifen-treated mice, demonstrating effective knockout of TRPC3 in SMCs. (**C**) Brain sections fluorescently immunolabeled against TRPC3 (purple) and smooth muscle alpha actin (green). Note the co-localization (white) of TRPC3 and smooth muscle alpha actin in small cerebral arteries of a littermate control and the absence of colocalization in cerebral arteries of a TRPC3smcKO mouse.

To isolate the contributions of TRPC3 channels to the IHR during SE and its impact on seizure progression, we developed a transgenic mouse line in which the calcium-permeable TRPC3 channels of smooth muscle cells are inducibly knocked out (Fig. 1A). Gel electrophoresis of PCR product from endothelium-denuded mesenteric arteries (Fig. 1B) provided evidence of a total knockout of TRPC3 channels in VSMCs. Co-localization of smooth muscle actin and TRPC3 (purple and green immunofluorescence; co-localization appears bright white) was apparent in brain sections from littermate controls, but was absent in analogous TRPC3smcKO sections (Fig. 1C). Collectively, these results confirm deletion of *TRPC3* gene expression in the VSMCs of our TRPC3smcKO mice.

To evaluate neurovascular coupling during SE, we simultaneously used laser speckle contrast imaging (LSI) to measure blood flow in one hemisphere and electroencephalography (EEG) to measure neural activity in the contralateral hemisphere. We previously reported that recording EEG from only one hemisphere is sufficient as the neural activity from one hemisphere is mirrored in the other (R = 0.98)^21^.

**Figure 2:**
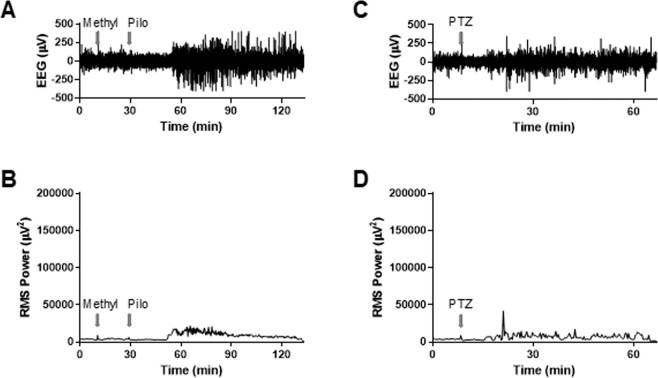
EEG analysis of the effect of pilocarpine or pentylenetetrazol on anesthetized mice (n = 3, each). (**A**) Representative raw EEG trace of WT mouse; arrows denote injection of methylscopolamine (Methyl; 10 mg/kg, i.p.) and pilocarpine (Pilo; 280 mg/kg, i.p.). (**B**) Root mean square (RMS) power analysis of EEG trace in panel A. (**C**) Representative raw EEG trace of WT mouse; arrow denotes injection of pentylenetetrazol (PTZ; 80 mg/kg, i.p.). (**D**) RMS power analysis of EEG trace in panel C.

**Figure 3:**
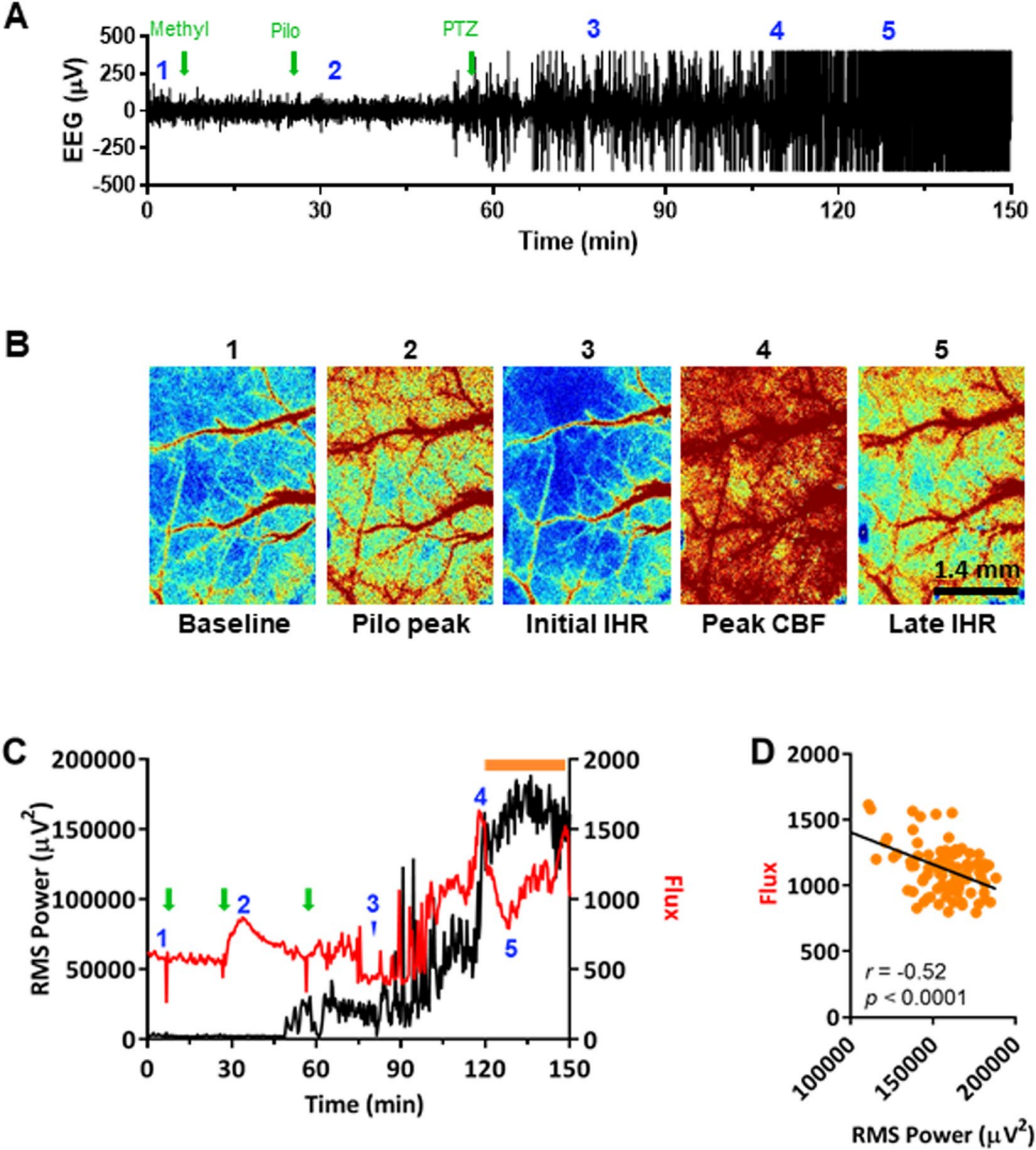
Effect of pilocarpine and pentylenetetrazol on neural activity and cerebral blood flow (CBF) in a representative anesthetized WT mouse as measured by simultaneous EEG and LSI. (**A**) Raw EEG trace demonstrating induction of SE by pilocarpine (pilo; 280 mg/kg, i.p.) and pentylenetetrazol (PTZ; 120 mg/kg, i.p.). Green arrows denote injection times; blue numbers denote acquisition time of each numbered LSI image in panel B. (**B**) LSI images of CBF at single time points. (**C**) RMS power and flux (CBF as measured by LSI) overlay demonstrates a period of severe IHR (orange bar). Green arrows and blue numbers carried over from panel A. (**D**) Analysis of late-SE CBF (Flux) and RMS power at the time period denoted by the orange bar in panel C reveal a negative correlation.

Induction of convulsive seizures in stereotactically immobilized mice necessitated anesthesia due to animal welfare concerns. Initial attempts to induce seizure in anesthetized mice with either pilocarpine (Fig. 2A,B) or pentylenetetrazol administration (Fig. 2C,D) failed as the neural activity after single administration of these chemical convulsants was far below the root mean square power of SE observed in unanesthetized mice^15^. However, we found that sequential administration of pilocarpine and pentylenetetrazol reliably induced seizures in anesthetized mice that were similar electrographically and behaviorally to the pilocarpine-induced seizures in unanesthetized mice (Fig. 3A).

Reports from clinical studies provide evidence of transient periods of hypoperfusion in response to different forms of acute brain insult, including SE^6,8–10^. In anesthetized wild-type mice, we observed what appeared to be a series of such IHRs shortly following the onset of seizure activity (Fig. 3B,C). Subsequently, as neural activity began to increase following pentylenetetrazol injection (Fig. 3A,C), there was a parallel increase in blood flow (Fig. 3C). This initial increase in blood flow was short-lived, however, as blood flow then sharply decreased, although neural activity remained high (i.e., an IHR). The initial IHR appeared to immediately precede the dramatic rise in measured neuronal activity marking the onset of SE. During the subsequent period of SE, blood flow intermittently increased but was highly variable, and throughout SE, additional IHRs occurred and blood flow failed to positively correlate with neural activity (Fig. 3D), demonstrating dysregulation of neurovascular coupling. To our knowledge, these experiments provide the first demonstration of seizure-induced IHR in an animal model.

**Figure 4:**
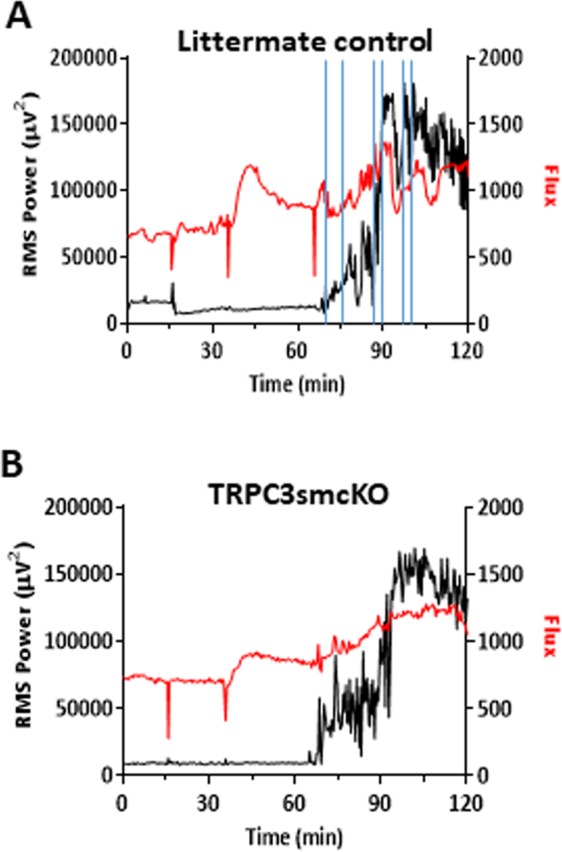
Analysis of neurovascular coupling by LSI/EEG. (**A**) RMS power/flux overlay of representative data from a littermate control mouse demonstrating IHR after SE onset. Periods of IHR are denoted between vertical blue lines. (**B**) RMS/flux overlay of representative data from a TRPC3smcKO mouse showing more sustained blood flow with less variability during late SE. The three sharp dips in the flux data are artifacts corresponding to the sequential injection of methylscopolamine, pilocarpine and pentylenetetrazol (as described in Fig. 3 legend), when the camera view was briefly occluded by the experimenter’s hand administering the injection. The camera was intentionally blocked to provide a clear marker on the flux trace of injection times.

**Figure 5:**
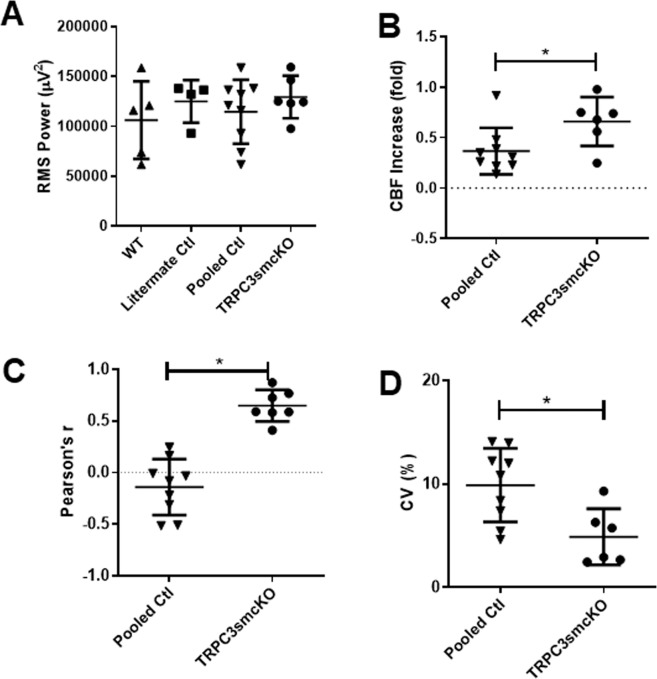
Analyses of neural activity and CBF recorded from TRPC3smcKO and pooled control mice. (**A**) RMS power analysis of EEG activity demonstrate no significant difference in seizure severity between TRPC3smcKO, WT, and littermate control mice. WT and littermate controls were pooled due to similarity. (**B**) Fold-change of CBF during SE, demonstrating a significant increase in blood flow of TRPC3smcKO mice compared with controls. (**C**) Plot of correlation between RMS power and CBF reveals a higher correlation in TRPC3smcKO mice compared to pooled controls. (**D**) Analysis of CBF variance during SE reveals significantly less CBF variability in TRPC3smcKO mice compared to controls. n = 5, 4, 9, 6 for WT, littermate controls, pooled controls, and TRPC3smcKO, respectively.

In order to examine the effect that genetic ablation of TRPC3 channels in cerebral VSMCs had on neurovascular coupling before and during SE, TRPC3smcKO mice and their genetically identical littermate controls treated with vehicle rather than tamoxifen, were tested as described for WT mice. As in WT mice, IHRs were observed in the littermate control mice (Fig. 4A). TRPC3smcKO mice exhibited reduced IHR, a more sustained increase in cerebral blood flow, and less variability during SE (Fig. 4B). Littermate control mice exhibited no significant differences from WT mice in seizure intensity, therefore WT and littermate controls were pooled (Fig. 5A). It is important to note that as seizure intensity was not significantly different between experimental groups (Fig. 5A), differences found in subsequent measures comparing neurovascular coupling between groups do not relate to seizure intensity. Compared with controls, TRPC3smcKO mice demonstrated a more sustained increase in cerebral blood flow during SE (0.66 ± 0.10 fold-change for TRPC3smcKO vs 0.37 ± 0.08 fold-change for pooled controls; Fig. 5B). During SE, control mice showed either a negative correlation or no correlation between blood flow and neural activity (−0.14 ± 0.09 Pearson’s r; Fig. 5C) whereas TRPC3smcKO mice consistently demonstrated a positive correlation (0.65 ± 0.06 Pearson’s r; Fig. 5C), indicating preservation of neurovascular coupling. TRPC3smcKO mice also demonstrated significantly less blood flow variability during SE (4.90 ± 1.11% for TRPC3smcKO vs 9.88 ± 1.19% for pooled controls; Fig. 5D). These data are consistent with the view that the features of SE-related blood flow dysfunction are largely absent in TRPC3smcKO mice and that IHR and disrupted neurovascular coupling are at least partly mediated by TRPC3 channels expressed in cerebral VSMCs.

**Figure 6:**
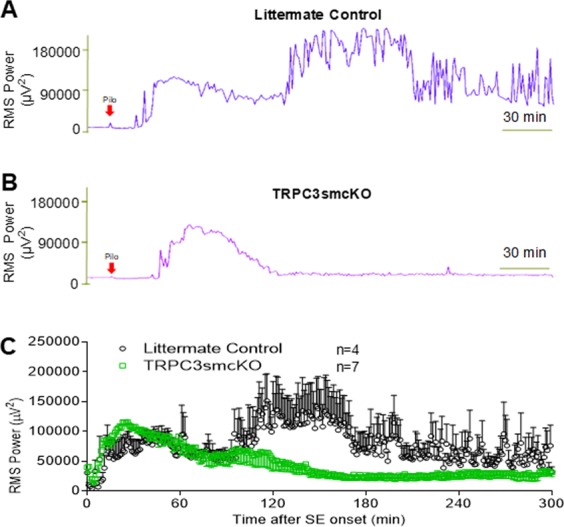
Pilocarpine-induced SE in TRPC3smcKO mice and littermate controls. (**A**) RMS power analysis of littermate controls demonstrates an initial phase of SE after pilocarpine injection followed by a higher magnitude second phase. (**B**) RMS power analysis of TRPC3smcKO mice shows only a brief initial phase of SE before return to near baseline activity. (**C**) Averaged RMS power values (aligned from SE onset) from 4 littermate control mice and 7 TRPC3smcKO mice demonstrate the lack of the second phase of SE in TRPC3smcKO mice.

In view of the reduction in seizure-induced IHR and improved neurovascular coupling during pilocarpine/pentylenetetrazol-induced SE in anesthetized TRPC3smcKO mice, we wanted to determine whether such protection of neurovascular coupling would impact the progression of SE in the standard pilocarpine model of SE in unanesthetized, freely-moving mice. If IHR is a critical contributor to SE progression, its amelioration in TRPC3smcKO mice would be predicted to reduce the intensity and/or duration of SE. Indeed, we show this to be the case in unanesthetized, pilocarpine-treated TRPC3smcKO mice (Fig. 6). Approximately 30 minutes after the onset of SE, both TRPC3smcKO and littermate controls showed a gradual reduction in neural activity over the next hour. Approximately 90 minutes after SE induction, the littermate controls exhibited a second increase in seizure activity that approximately doubled the root mean square power of the first phase. Interestingly, this second phase of SE was completely absent in TRPC3smcKO mice, in which there was a continued and steady reduction of neural activity and seizures. Thus, ablation of smooth muscle TRPC3 channels reduces both duration and severity of pilocarpine-induced seizure activity in TRPC3smcKO mice compared to littermate controls.

## Discussion

In this study, we demonstrated that wild-type and littermate control mice show cerebrovascular dysfunction during SE, whereas TRPC3smcKO mice show preserved neurovascular coupling. Importantly, littermate control mice showed a prolonged pilocarpine-induced SE with two distinct phases whereas TRPC3smcKO mice showed a significant reduction in pilocarpine-induced SE duration that was due to the complete absence of the second phase of SE observed in littermate controls. These observations suggest that TRPC3 channels in cerebral VSMCs mediate the cerebrovascular dysfunction induced by SE and that TRPC3-mediated cerebrovascular dysfunction is an important contributor to the progression of SE.

Clinical studies show that acute insults to the brain, including the intense and sustained seizure activity of SE, are associated with an IHR, which almost certainly exacerbates neuronal dysfunction and likely contributes to the progression of SE^6,10^. However, the IHR has not been demonstrated in an animal model of seizure to our knowledge. In the current study, we addressed this initial problem by developing a new seizure model using sequential administration of the chemical convulsants, pilocarpine and pentylenetetrazol (Fig. 2A,B). Our model allows for the first reliable assessment of the IHR in an animal model of seizure and opens the door to preclinical opportunities to explore mechanisms of IHR and evaluate the benefit of new therapeutics.

In contrast to WT and littermate control mice, TRPC3smcKO mice demonstrated a reduction in the occurrence and severity of IHRs and showed increased blood flow during SE that remained stable during the experiment and was positively correlated with neural activity, thereby demonstrating preserved neurovascular coupling. We interpret this finding as evidence that TRPC3 channels in cerebral VSMCs mediate a pathogenic dysregulation of neurovascular coupling during seizure, including the inappropriate vasoconstriction that is the key feature of IHR, and they are at least partially responsible for the reduced and inconsistent blood supply during seizure. The mismatch between neuronal activity and cerebral blood flow is tantamount to functional hypoxia, which would be expected to trigger the complex signaling cascades leading to neuroinflammation and neuronal cell death, which have been documented in the pilocarpine-induced SE model^22,23^.

Because the TRPC3 channel is expressed in many cell types and its functions are understood to be cell-specific^24^, our SMC-specific TRPC3 knockout mouse constitutes a valuable tool for determining cell-specific roles for TRPC3 channels in pathological impairment of neurovascular coupling in epilepsy, stroke, traumatic brain injury, and other neurological conditions. However, as smooth muscle cells are expressed throughout the body in arteries, veins, and various organs, it is important to note that in our animal model, TRPC3 is ablated not only in cerebral VSMCs but also in other smooth muscle cells. Although the peripheral deletion of TRPC3 channels appears unlikely to result in changes of neurovascular coupling, this possibility cannot be completely ruled out. Additionally, “leaky Cre” or limited off-target Cre expression has been reported in some inducible Cre mouse lines^25^. The smMHC-Cre line used in the present study carries the estrogen receptor mutation, which is widely used since it minimizes leaky expression of *Cre*^19,25–30^. However, lacking a comprehensive analysis of different cell types in our TRPC3smcKO mouse model, we cannot definitely conclude that *Cre* expression was limited solely to smooth muscle cells.

In the pilocarpine model of freely-moving un-anesthetized mice, LSI was unable to be employed, precluding measurements of cerebral blood flow. Thus, we cannot state definitively that the IHR occurs in un-anesthetized mice, or contributes to the intensification and prolongation of seizure in the pilocarpine-only model of SE. However, we infer from the presence of IHR as a consistent feature in WT and littermate control mice in the anesthetized pilocarpine/pentylenetetrazol model of seizure, that IHR may be a feature of the pilocarpine-only model of seizure in these same animals. This inference also is supported by clinical reports documenting the IHR during SE in human subjects^10^, and during other insults to the brain^8^. Thus, we suggest that the observation of IHR during SE in our study is not likely an artifact of a particular pharmacological treatment. We also reason that because IHR is significantly reduced and cerebral blood flow improved in TRPC3smcKO mice compared to control animals, this preservation of neurovascular coupling is likely the mechanism by which pilocarpine-induced SE in these mice is ameliorated so dramatically compared to littermate controls.

Collectively, our results suggest that TRPC3 channels expressed by cerebral VSMCs mediate a pathogenic vasoconstrictive response during sustained seizure activity. This response is observed as an IHR and a subsequent dysregulation of neurovascular coupling, which appears to increase the intensity and duration of SE. The finding that VSMC-expressed TRPC3 channels contribute to seizure-induced IHR may have broad implications not only for seizure disorders, but also for other neurological disorders with a component of neurovascular dysfunction^9^.

## Methods

### Ethical approval

This study was carried out in strict accordance with the recommendations in the Guide for the Care and Use of Laboratory Animals of the National Institutes of Health. The protocol was approved by Institutional Animal Use and Care Committee at the University of Arkansas for Medical Sciences. All surgery was performed under urethane and sevoflurane anesthesia, and all efforts were made to minimize suffering. Mice were group housed under a 12-hour light/dark cycle with food and water ad libitum.

### Smooth muscle cell-specific TRPC3 knockout mice

Smooth muscle-specific TRPC3 knockout (TRPC3smcKO) mice were generated by crossing TRPC3flx/flx mice^20^ with a well-characterized Cre line expressing tamoxifen-inducible Cre recombinase under control of a smooth muscle cell myosin heavy chain (smMHC-Cre) promoter^19^. The ion-conducting pore-forming region of the TRPC3 channel is encoded by exon 7 of the *TRPC3* gene and is flanked by introduced loxP sites in TRPC3flx/flx mice. Thus, expression of Cre recombinase results in the loss of exon 7 and ablation of functional TRPC3 channels. Smooth muscle cell-specific knockout of TRPC3 was induced by injection of tamoxifen emulsified in corn oil (1 or 2 mg/100 μL, i.p.) once daily for 5 consecutive days. Mice were used between 9 days and 13 days after the last injection. Littermate control mice were generated from the same line by once daily injection of 100 μL vehicle (corn oil) for 5 consecutive days.

### Immunohistochemistry double labeling

Mice were transcardially perfusion-fixed, and brains were post-fixed in 4% paraformaldehyde in phosphate buffer for 24 hours. Fixed brains were washed in phosphate buffered saline, then cryopreserved by immersion in 15% sucrose for 24 hours followed by 30% sucrose for 24 hours. Brains were frozen in O.C.T. compound (Sakura Finetek) at −80 °C, sliced on a cryostat (Microm HM-525; Thermo Scientific) into 20-μm sections, and adhered to Fisher Superfrost slides (Fisher Scientific). Alpha-smooth muscle actin monoclonal antibody (Invitrogen Cat# 14-9760-82, Lot# 2033287, RRID: AB_2572996)^31^ was used at 1:200 dilution with the Vector mouse-on-mouse fluorescein kit (Vector Labs Cat# FMK-2201, Lot# ZF0426, RRID:AB_2336834) and a knock out-validated rabbit anti-mouse TRPC3 polyclonal antibody (Alomone Labs Cat# ACC016, Lot# ACC016AG1240, RRID:AB_2040236)^32^ was used at 1:400 dilution with goat anti-rabbit Alexa 647-conjugated secondary antibody (Jackson ImmunoResearch Labs Cat# 111-606-144, RRID:AB_2338083) at 1:500 dilution. Coverslips were mounted with ProLong Gold Antifade Mountant with Dapi (Life Technologies).

### Combined EEG and LSI

Age-matched male TRPC3smcKO mice, littermate controls, and wild type (WT) mice were anesthetized with urethane (1.1 mg/kg, i.p.) and placed in a small animal stereotactic instrument (Kopf Instruments). Rectal temperature was maintained at 37 °C during the entire experiment with the use of a heating pad as ensured by a homeothermic monitor (Harvard Apparatus). Sevoflurane was used during surgery to augment urethane anesthesia and was administered at 3% by volume and 2.0 L/min oxygen using a veterinary sevoflurane vaporizer (VetEquip). Sevoflurane was chosen instead of isoflurane because sevoflurane preserves autoregulation better than isoflurane^33^. An OmniDrill 35 with a silicon carbide abrasive tip (World Precision Instruments) was used to thin the parietal bone over the left hemisphere from suture to suture until transparency, thus creating a non-invasive, rectangular “window” of approximately 3 × 2 mm to increase imaging resolution. Two recording electrodes were inserted through the skull to touch the dura overlying the right hemisphere. The front electrode was placed over the motor cortex (approximately 1 mm anterior to bregma and 1.5 mm lateral to midline). The other electrode was placed approximately 1.5 mm anterior to lambda and 1.5 mm lateral to the midline. The ground electrode was placed in the midline approximately 1.5 mm posterior of lambda, over the cerebellum^21^. After completion of surgery, sevoflurane administration was discontinued for the remainder of the experiment while oxygen administration was maintained. A laser speckle contrast imager was then placed over the transparent section of skull and focused to image the cortical surface and subsurface vasculature, and EEG and LSI recordings were begun.

### Pilocarpine/pentylenetetrazol model of status epilepticus in anesthetized mice

To induce seizures in anesthetized mice, we modified the widely used pilocarpine and pentylenetetrazol models of SE. Mice were administered a single dose of methylscopolamine nitrate (10 mg/kg, i.p.) to block the peripheral effects of pilocarpine^34,35^, followed approximately 20 minutes later by a single dose of pilocarpine (280 mg/kg, i.p.). Finally, a single dose of pentylenetetrazol (120 mg/kg, i.p.) was administered after another 30 minutes.

### Pilocarpine-induced seizures and EEG recording

EEG head-mount surgery of age-matched (2.8 to 3-month-old) TRPC3smcKO and littermate control male mice was conducted as described previously^15^. Mice were administered a single dose of methylscopolamine nitrate (10 mg/kg, i.p.) to block the peripheral effects of pilocarpine, followed approximately 20 minutes later by a single dose of pilocarpine (280 mg/kg, i.p.). Pilocarpine-induced seizures were recorded using the Pinnacle 8200 recording system (Pinnacle Technology) as described previously^21^.

### EEG root mean square power analysis

Fast Fourier power spectral analysis (FFT) of EEG signals was performed using Sirenia Seizure Pro software (Pinnacle Technology) with a Hanning window applied to reduce spectral leakage. The full frequency bandwidth was set at 0–1000 Hz. The root mean square power values for the full frequency band were calculated in one minute intervals after pilocarpine administration by averaging the values of 15 four-second windows.

### Laser speckle image analysis and determination of IHR

A Moor FLPI-2 laser speckle contrast imager was used to generate laser speckle video of mouse cerebral blood flow. The laser wavelength was 785 nm, camera resolution was 580 × 752 pixels, and cell size for spatial image processing was 5 × 5 pixels. The exposure time was 20 ms and temporal resolution was 4 seconds per frame. Laser speckle video was analyzed with Moor FLPI-2 Review V5.0 software (Moor instruments). A region of interest (ROI) was drawn inside the thinned “skull window”, and the mean flux (blood flow) value was calculated from the ROI. The skull window was a rounded, irregular rectangle following the shape of the parietal bone as delimited by the coronal, sagittal, and lambdoid sutures. There were subtle variations in parietal bone shape, size, and suture thickness between animals which had a very slight impact on window shape. Therefore, we chose to use the software’s ellipse tool in order to draw uniform ROI that were highly similar from animal to animal rather than drawing ROI free-hand to follow the shape of each individual window, which would result in greater variation of ROI between animals. Unavoidably, an ellipse drawn inside a rectangle cannot contain the entirety of the area of the rectangle, but the ellipse was drawn to include as much of the rectangular window area as possible, containing approximately two thirds of the total window area. To determine whether there was a difference in response between the apparent vessels within the ROI and the surrounding regions vascularized by capillaries, we drew regions of interest specifically excluding the large surface vessels. Flux changes in ROI that excluded the large surface vessels paralleled that of ROI that included them. The measurement period for baseline flux was five minutes immediately preceding the first injection (methylscopolamine). Analysis of blood flow during SE (Fig. 5) included the period of SE corresponding to the RMS plateau. IHR is not defined as a reduction in CBF, but rather as a period of hypoperfusion during which CBF is inappropriately low relative to the current level of neuronal activity.

### Statistics

Littermates were randomly assigned to either the tamoxifen-treated (TRPC3smcKO) or vehicle-treated (littermate control) group. Experimenters were not blind to animal treatment conditions. To discern whether ablation of smooth muscle cell TRPC3 channels ameliorates cerebrovascular dysfunction in SE, we first performed an ANOVA on seizure intensity (Fig. 5A; WT n = 5, littermate controls n = 4, pooled controls n = 9, TRPC3smcKO n = 6). Since there was no significant difference between WT and littermate controls, they were pooled for the remaining analyses. Next, we performed unpaired t-tests between pooled controls and TRPC3smcKO mice to determine whether there was a significant difference in cerebral blood flow during SE (Fig. 5B), in Pearson’s correlation factors (Fig. 5C), and/or in coefficient of variance (Fig. 5D). Two-way ANOVA was used to compare SE intensity between littermate controls and TRPC3smcKO mice (Fig. 6). Data in Figs. 5 and 6 are shown as mean ± standard deviation (SD). P values of 0.05 or less (p < 0.05) were considered statistically significant. All data sets passed the normality test for distribution.

## Supplementary information

Supplementary Dataset 1.

## Data Availability

The datasets generated during and/or analyzed during the current study are available from the corresponding author on reasonable request.

## References

[1] Trinka E, Höfler J, Zerbs A (2012). Causes of status epilepticus. Epilepsia.

[2] Lo EH, Dalkara T, Moskowitz MA (2003). Mechanisms, challenges and opportunities in stroke. Nat. Rev. Neurosci..

[3] Iadecola C (2004). Neurovascular regulation in the normal brain and in Alzheimer’s disease. Nat. Rev. Neurosci..

[4] Stanimirovic DB, Friedman A (2012). Pathophysiology of the Neurovascular Unit: Disease Cause or Consequence?. J. Cereb. Blood Flow Metab..

[5] Iadecola C (2017). The Neurovascular Unit Coming of Age: A Journey through Neurovascular Coupling in Health and Disease. Neuron.

[6] Winkler MKL (2012). Impaired neurovascular coupling to ictal epileptic activity and spreading depolarization in a patient with subarachnoid hemorrhage: Possible link to blood-brain barrier dysfunction. Epilepsia.

[7] Kovács R, Heinemann U, Steinhäuser C (2012). Mechanisms underlying blood-brain barrier dysfunction in brain pathology and epileptogenesis: Role of astroglia. Epilepsia.

[8] Hinzman JM (2014). Inverse neurovascular coupling to cortical spreading depolarizations in severe brain trauma. Brain.

[9] Dreier JP (2011). The role of spreading depression, spreading depolarization and spreading ischemia in neurological disease. Nat. Med..

[10] Dreier JP (2012). Spreading convulsions, spreading depolarization and epileptogenesis in human cerebral cortex. Brain.

[11] Warren CP (2010). Synchrony in normal and focal epileptic brain: the seizure onset zone is functionally disconnected. J. Neurophysiol..

[12] Kim D-S, Ryu HJ, Kim J-E, Kang T-C (2013). The reverse roles of transient receptor potential canonical channel-3 and -6 in neuronal death following pilocarpine-induced status epilepticus. Cell. Mol. Neurobiol..

[13] Ryu HJ (2013). Endothelial transient receptor potential conical channel (TRPC)-3 activation induces vasogenic edema formation in the rat piriform cortex following status epilepticus. Cell. Mol. Neurobiol..

[14] Zeng C (2015). Upregulation and Diverse Roles of TRPC3 and TRPC6 in Synaptic Reorganization of the Mossy Fiber Pathway in Temporal Lobe Epilepsy. Mol. Neurobiol..

[15] Phelan Kevin D., Shwe U Thaung, Cozart Michael A., Wu Hong, Mock Matthew M., Abramowitz Joel, Birnbaumer Lutz, Zheng Fang (2016). TRPC3 channels play a critical role in the theta component of pilocarpine-induced status epilepticus in mice. Epilepsia.

[16] Hill AJ (2006). A TRPC-like non-selective cation current activated by α1-adrenoceptors in rat mesenteric artery smooth muscle cells. Cell Calcium.

[17] Adebiyi A (2010). Isoform-Selective Physical Coupling of TRPC3 Channels to IP 3 Receptors in Smooth Muscle Cells Regulates Arterial Contractility. Circ. Res..

[18] Adebiyi A (2012). An Elevation in Physical Coupling of Type 1 Inositol 1,4,5-Trisphosphate (IP 3) Receptors to Transient Receptor Potential 3 (TRPC3) Channels Constricts Mesenteric Arteries in Genetic Hypertension. Hypertension.

[19] Regan CP, Manabe I, Owens GK (2000). Development of a Smooth Muscle–Targeted Cre Recombinase Mouse Reveals Novel Insights Regarding Smooth Muscle Myosin Heavy Chain Promoter Regulation. Circ. Res..

[20] Hartmann J (2008). TRPC3 channels are required for synaptic transmission and motor coordination. Neuron.

[21] Phelan KD, Shwe UT, Williams DK, Greenfield LJ, Zheng F (2015). Pilocarpine-induced status epilepticus in mice: A comparison of spectral analysis of electroencephalogram and behavioral grading using the Racine scale. Epilepsy Res..

[22] Dingledine R, Varvel NH, Dudek FE (2014). When and how do seizures kill neurons, and is cell death relevant to epileptogenesis?. Adv. Exp. Med. Biol..

[23] Varvel NH (2016). Infiltrating monocytes promote brain inflammation and exacerbate neuronal damage after status epilepticus. Proc. Natl. Acad. Sci. USA.

[24] Eder, P., Poteser, M. & Groschner, K. TRPC3: a multifunctional, pore-forming signalling molecule. *Handb. Exp. Pharmacol*. 77–92 (2007). 10.1007/978-3-540-34891-7_4 10.1007/978-3-540-34891-7_4 17217051

[25] Hartmann DA, Underly RG, Watson AN, Shih AY (2015). A murine toolbox for imaging the neurovascular unit. Microcirculation.

[26] Xin H-B, Deng K-Y, Rishniw M, Ji G, Kotlikoff MI (2002). Smooth muscle expression of Cre recombinase and eGFP in transgenic mice. Physiol. Genomics.

[27] Lounev VY (2009). Identification of progenitor cells that contribute to heterotopic skeletogenesis. J. Bone Joint Surg. Am..

[28] Mao X (2012). Vascular smooth muscle cell Smad4 gene is important for mouse vascular development. Arterioscler. Thromb. Vasc. Biol..

[29] Youn J-Y (2014). Role of vascular oxidative stress in obesity and metabolic syndrome. Diabetes.

[30] Ustiyan V (2018). FOXF1 transcription factor promotes lung morphogenesis by inducing cellular proliferation in fetal lung mesenchyme. Dev. Biol..

[31] Chaponnier C, Gabbiani G (2016). Monoclonal antibodies against muscle actin isoforms: epitope identification and analysis of isoform expression by immunoblot and immunostaining in normal and regenerating skeletal muscle. F1000Research.

[32] Mizoguchi Y (2014). Brain-derived Neurotrophic Factor (BDNF) Induces Sustained Intracellular Ca 2+ Elevation through the Up-regulation of Surface Transient Receptor Potential 3 (TRPC3) Channels in Rodent Microglia. J. Biol. Chem..

[33] Summors AC, Gupta AK, Matta BF (1999). Dynamic cerebral autoregulation during sevoflurane anesthesia: a comparison with isoflurane. Anesth. Analg..

[34] Burke K, Chandler CJ, Starr BS, Starr MS (1990). Seizure promotion and protection by D-1 and D-2 dopaminergic drugs in the mouse. Pharmacol. Biochem. Behav..

[35] Priel MR, dos Santos NF, Cavalheiro EA (1996). Developmental aspects of the pilocarpine model of epilepsy. Epilepsy Res..

