# Supplementary material for: Vascular smooth muscle TRPC3 channels facilitate the inverse hemodynamic response during status epilepticus
Source: Sci Rep. 2020 Jan 21;10:812. doi: 10.1038/s41598-020-57733-0 (PMC6972937; doi:10.1038/s41598-020-57733-0)
Supplement: Supplementary file 1 — Supplementary Dataset 1. [file 41598_2020_57733_MOESM1_ESM.pdf]

Vascular smooth muscle TRPC3 channels facilitate the inverse hemodynamic response during status epilepticus

Michael A. Cozart,[1]* Kevin D. Phelan,[2] Hong Wu,[1] Shengyu Mu,[1] Lutz Birnbaumer,[3,4] Nancy J. Rusch,[1] Fang Zheng[1]

[1]Department of Pharmacology and Toxicology, [2]Department of Neurobiology and Developmental Sciences, University of Arkansas for Medical Sciences, Little Rock, Arkansas, United States of America

[3]Neurobiology Laboratory, National Institute of Environmental Sciences, Research Triangle Park, North Carolina, United States of America

[4]Institute of Biomedical Research (BIOMED), School of Medical Sciences, Catholic University of Argentina, Buenos Aires, Argentina.

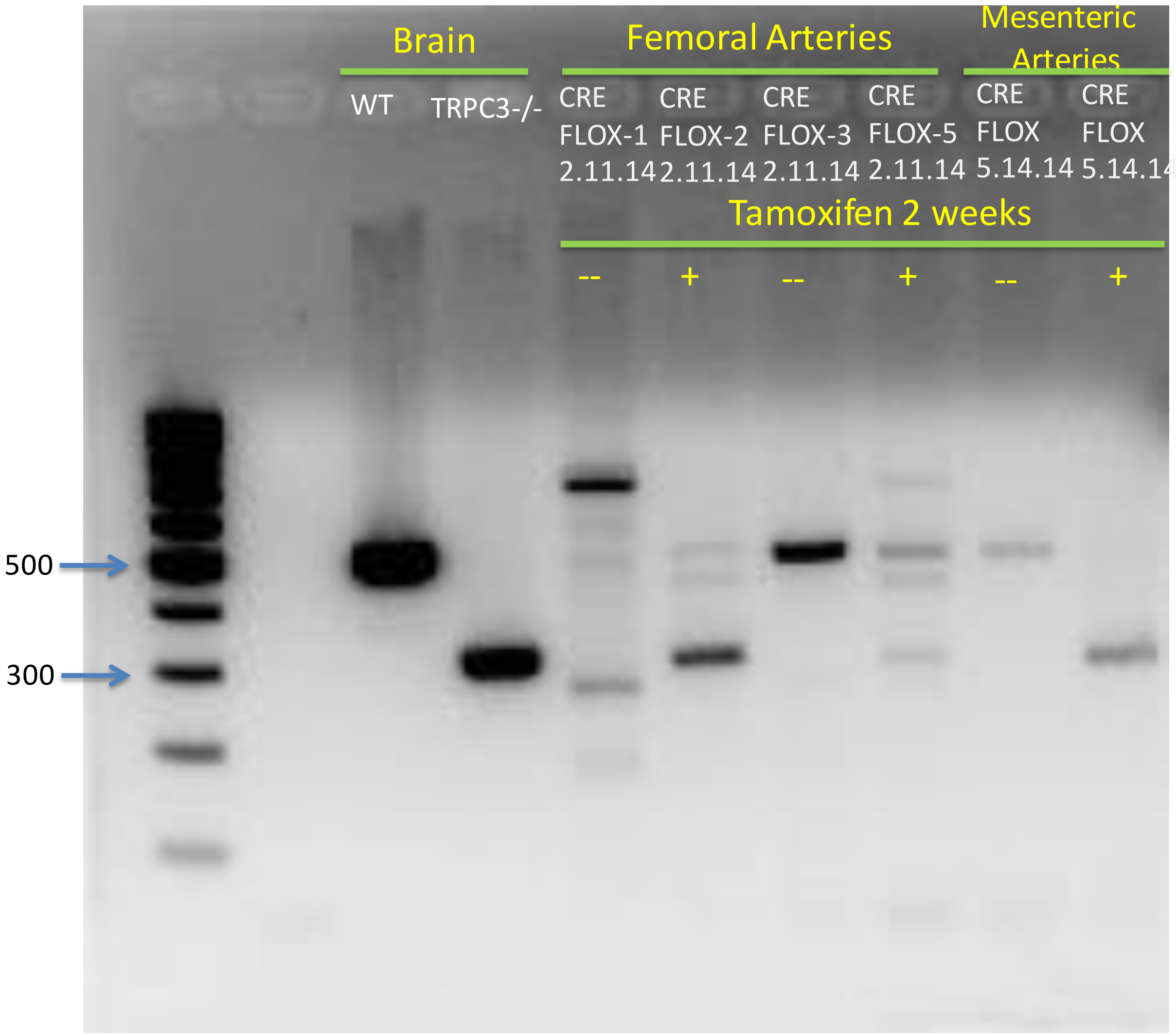

Brain
Femoral Arteries
Mesenteric Arteries
WT
TRPC3-/-
CRE FLOX-1 2.11.14
CRE FLOX-2 2.11.14
CRE FLOX-3 2.11.14
CRE FLOX-5 2.11.14
CRE FLOX 5.14.14
CRE FLOX 5.14.14
Tamoxifen 2 weeks
--
+
--
+
--
+
500
300
